# High‐Performance Li–O_2_ Batteries with Controlled Li_2_O_2_ Growth in Graphene/Au‐Nanoparticles/Au‐Nanosheets Sandwich

**DOI:** 10.1002/advs.201500339

**Published:** 2016-04-28

**Authors:** Guoqing Wang, Fangfang Tu, Jian Xie, Gaohui Du, Shichao Zhang, Gaoshao Cao, Xinbing Zhao

**Affiliations:** ^1^ State Key Laboratory of Silicon MaterialsSchool of Materials Science and EngineeringZhejiang UniversityHangzhou310027P. R. China; ^2^ Key Laboratory of Advanced Materials and Applicationsfor Batteries of Zhejiang ProvinceHangzhou310027P. R. China; ^3^ Institute of Physical ChemistryZhejiang Normal UniversityJinhua321004P. R. China; ^4^ School of Materials Science and EngineeringBeijing University of Aeronautics and AstronauticsBeijing100191P. R. China

**Keywords:** catalysis, controlled growth, Li_2_O_2_, Li–O_2_ batteries, sandwich

## Abstract

The working of nonaqueous Li–O_2_ batteries relies on the reversible formation/decomposition of Li_2_O_2_ which is electrically insulating and reactive with carbon and electrolyte. Realizing controlled growth of Li_2_O_2_ is a prerequisite for high performance of Li–O_2_ batteries. In this work, a sandwich‐structured catalytic cathode is designed: graphene/Au‐nanoparticles/Au‐nanosheets (G/Au‐NP/Au‐NS) that enables controlled growth of Li_2_O_2_ spatially and structurally. It is found that thin‐layer Li_2_O_2_ (below 10 nm) can grow conformally on the surface of Au NPs confined in between graphene and Au NSs. This unique crystalline behavior of Li_2_O_2_ effectively relieves or defers the electrode deactivation with Li_2_O_2_ accumulation and largely reduces the contact of Li_2_O_2_ with graphene and electrolyte. As a result, Li–O_2_ batteries with the G/Au‐NP/Au‐NS cathode exhibit superior electrochemical performance. A stable cycling of battery can last 300 times at 400 mA g^−1^ when the capacity is limited at 500 mAh g^−1^. This work provides a practical design of catalytic cathodes capable of controlling Li_2_O_2_ growth.

## 1 Introduction

An increasing importance has been attached to the electrified transport to meet ever pressing energy and environmental issues. Although vehicles have now been powered by Li‐ion batteries (LIBs), the driving range of electric vehicles, however, is limited by low energy density of current LIBs.^1^, ^2^, ^3^ The emergence of Li–air (or Li–O_2_) batteries provides an appealing solution to this problem since it can deliver a theoretical energy density of 3505 Wh kg^−1^ by the reaction 2Li^+^ + 2e^−^ + O_2_ ↔ Li_2_O_2_, remarkably higher than that of current LIBs.^3^, ^4^, ^5^, ^6^, ^7^, ^8^, ^9^, ^10^, ^11^, ^12^, ^13^, ^14^, ^15^ Despite recent advancements, great challenges still maintain to develop practical Li–air batteries because the intrinsically low electrical conductivity of Li_2_O_2_ renders sluggish oxygen reduction/evolution reaction (ORR/OER).^16^, ^17^, ^18^ Generally, the ORR and OER kinetics can be considerably enhanced by using efficient catalysts,^19^, ^20^, ^21^, ^22^, ^23^, ^24^, ^25^ even though the efficiency of the catalysts is still questionable.^26^

It is widely accepted that noble metals have the best electrocatalytic activity for ORR and OER in organic systems. The work by Lu et al. showed that both charge and discharge overpotentials of Li–O_2_ batteries could be obviously reduced by using a bifunctional Pt/Au catalyst, where Pt and Au catalyze OER and ORR, respectively.^27^ Peng et al. found that Li–O_2_ battery can retain 95% of its capacity after 100 cycles with a low‐charge overpotential by using a nanoporous gold cathode.^28^ Recent reports have shown that Li–O_2_ batteries with noble metal catalysts, such as Pd,^29^, ^30^, ^31^ Ru,^32^, ^33^, ^34^, ^35^ and Pt,^36^, ^37^, ^38^, ^39^ exhibited low overpotentials and long‐term cycling stability. To prepare noble‐metal‐based catalysts, a carbon material is usually needed to support the noble metals. Unfortunately, carbon materials suffer from decomposition in the presence of Li_2_O_2_ or LiO_2_,^40^, ^41^, ^42^ especially those with defects.^43^ In addition, carbon, particularly that contains defects, also catalyzes the electrolyte decomposition during cycling.^30^, ^41^ Furthermore, polymer binders are chemically/electrochemically unstable in contact with Li_2_O_2_ or LiO_2_.^44^, ^45^, ^46^

Many strategies have been proposed to overcome the above problems related to the reactive Li_2_O_2_ or LiO_2_. One of the effective methods to alleviate the side reactions is to prepare carbon and/or binder‐free electrodes.^35^, ^47^, ^48^, ^49^, ^50^, ^51^, ^52^, ^53^, ^54^, ^55^, ^56^, ^57^ Chang et al. prepared a carbon/binder‐free RuO*_x_*/TiN nanotube arrays cathode, which exhibited an excellent cycling stability over 300 cycles.^35^ For practical applications, however, carbon matrices are sometimes necessary to ensure good electronic conductivity of the electrodes and decrease the usage amount of noble metals. In this regard, a modification on carbon materials is required to minimize the detrimental effects. Lu et al. provided a useful way to deactivate the active carbon defect sites through an alumina coating.^30^ The active sites on carbon could also be deactivated by in situ electrochemical nitrogen doping.^58^ The introduction of a component that preferably reacts with Li_2_O_2_ or LiO_2_ over carbon has proven to be an effective measure to lessen carbon‐induced negative effects.^59^

In this work, we provide a unique design of binder‐free catalytic cathode which was prepared in ice bath. In this cathode, Au nanoparticles (Au‐NP) are sandwiched between few‐layer graphene (G) and thin Au nanosheets (Au‐NS), forming a G/Au‐NP/Au‐NS sandwich frame. The merits of the electrode design include: (1) graphene provides the electronically conducting channels for ORR and OER; (2) Au NPs catalyze the confined/conformal growth of Li_2_O_2_ only on the surface of Au particles; (3) Au NSs fix the Au particles and encapsulate Li_2_O_2_‐loaded Au NPs between the sandwich frame. This unique electrode design effectively alleviates or defers the deactivation of the electrode and reduces the contact of Li_2_O_2_ (or LiO_2_) with graphene and electrolyte. As a result, Li–O_2_ batteries with the G/Au‐NP/Au‐NS cathode can sustain 300 cycles under 400 mA g^−1^ at a limited capacity of 500 mAh g^−1^. The Li–O_2_ battery can also sustain 100 cycles at a full charge/discharge mode in the cutoff voltage of 2–4.5 V. This work sheds light on the design of efficient catalytic cathodes enabling controlled Li_2_O_2_ growth aiming at high‐performance Li–O_2_ batteries.

## 2 Results and Discussion

**Figure 1:**
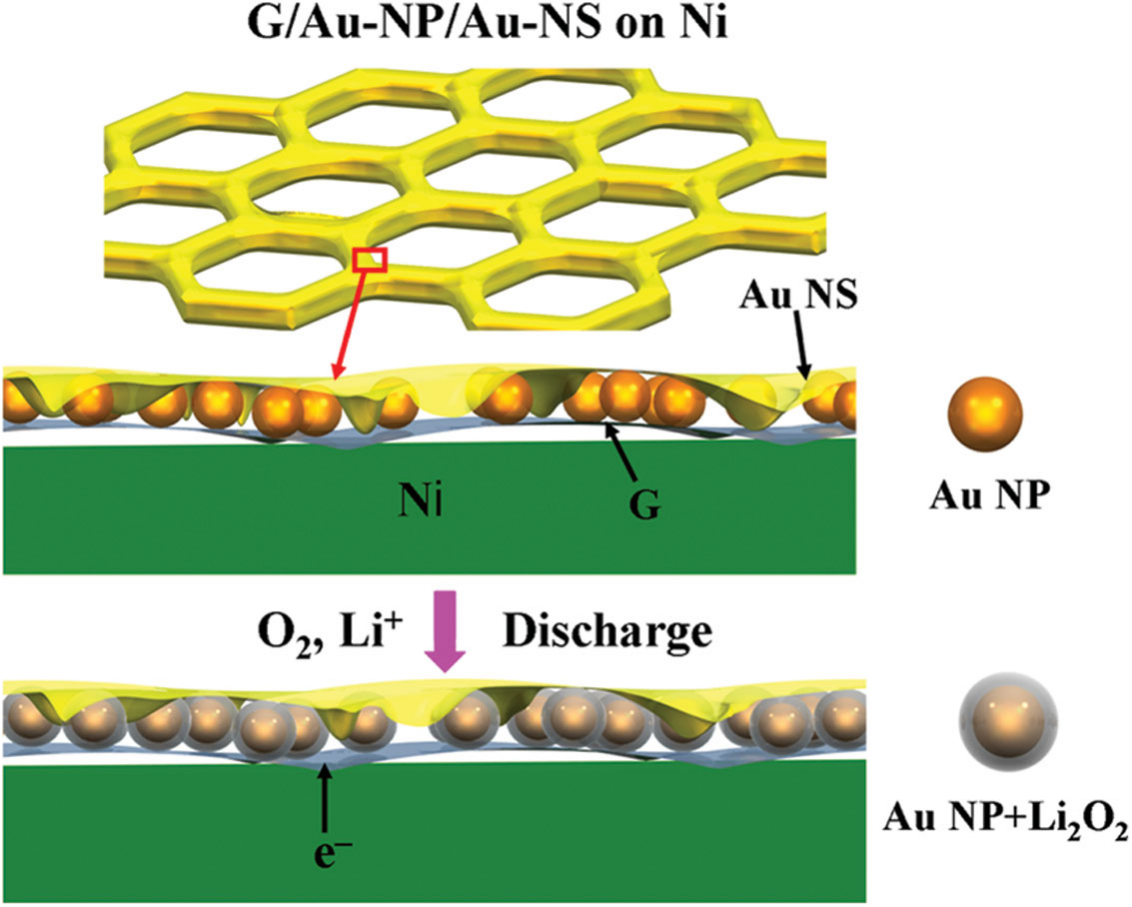
Schematic illustration of G/Au‐NP/Au‐NS electrode on Ni and its working mechanism.

**Figure** **1** shows the architecture and working mechanism of the G/Au‐NP/Au‐NS electrode, where Au NPs are encapsulated in between graphene and Au NSs. For the electrode, graphene layer was deposited on Ni foam substrate by chemical vapor deposition (CVD) method, and the Au‐NP/Au‐NS layer was deposited on graphene by solution impregnation method in ice bath. Two forms of nanosized Au, NPs and NSs, can grow simultaneously on graphene in ice bath. The Au NPs may catalyze the growth of Au NSs at low temperature. Li_2_O_2_ can realize the confined/conformal growth within the electrode, namely, only on the surface of Au NPs.

**Figure 2:**
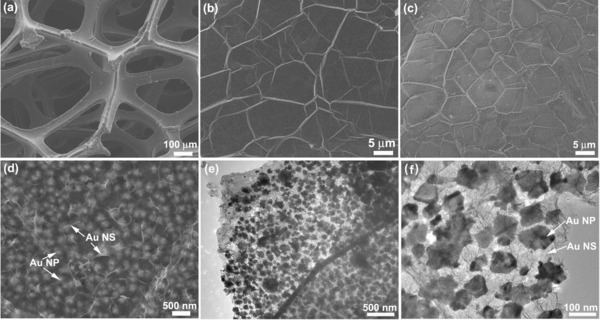
a) SEM image of graphene on Ni foam, b) enlarged view of (a), c) SEM image of the pristine G/Au‐NP/Au‐NS electrode on Ni, d) enlarged view of (c), e) TEM image of Au‐NP/Au‐NS exfoliated from the pristine G/Au‐NP/Au‐NS electrode, and f) enlarged view of (e).

**Figure** **2**a presents the scanning electron microscopy (SEM) image of porous Ni foam coated with graphene. Graphene was coated only on the skeleton of Ni foam and the porous structure of Ni is preserved for barrier‐free Li‐ion and oxygen transport. The enlarged view in Figure 2b suggests that the deposited graphene is in a thin‐layer form since the surface profile of Ni can be clearly seen. The 2D characteristics peak on Raman spectrum in Figure S1a (Supporting Information) verifies the few‐layer feature of the graphene.^60^ The surface profile of Ni can still be seen after Au deposition, suggesting that the Au layer on graphene is rather thin. The magnified view in Figure 2d exhibits that the Au layer consists of Au NPs and the Au NSs. Transmission electron microscopy (TEM) images in Figure 2e,f show that the size of Au NPs is around 100 nm. The presence of Au is confirmed by X‐ray diffraction (XRD, Figure S1c, Supporting Information), X‐ray photoelectron spectrum (XPS, Figure S1b, Supporting Information), and high‐resolution TEM (HRTEM, Figure S2, Supporting Information). The thickness of the Au NSs is estimated to below 2 nm from the folded domain of the Au sheets (Figure S2b, Supporting Information). As a result, a G/Au‐NP/Au‐NS sandwich has constructed by the solution impregnation method in ice bath. In contrast, only Au NPs form on graphene (G/Au‐NP) when the impregnation step was performed at room temperature (Figure S3a, Supporting Information).

**Figure 3:**
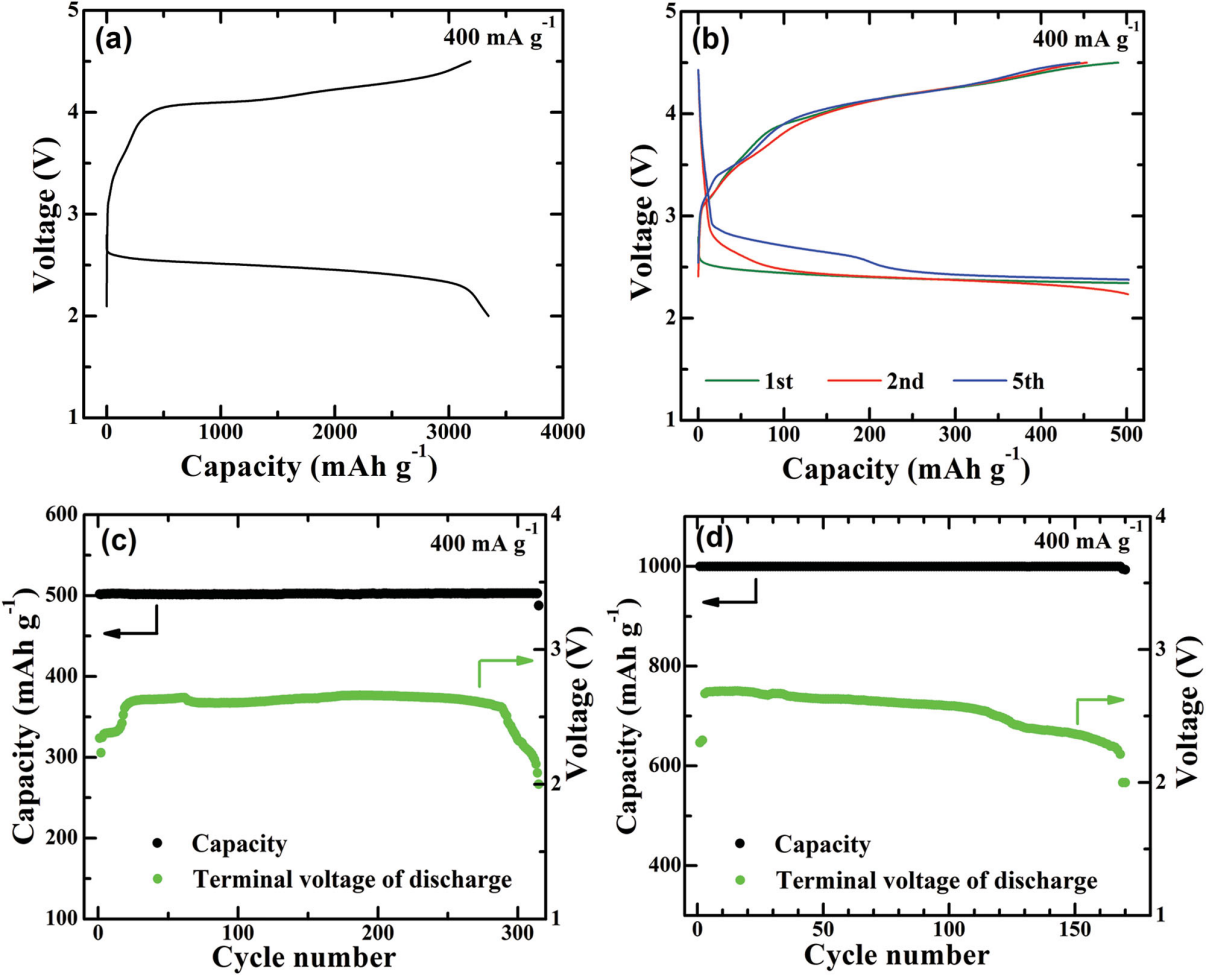
Electrochemical performance of Li–O_2_ batteries with the G/Au‐NP/Au‐NS cathode at a current density of 400 mA g^−1^: a) voltage profiles at a cutoff voltage of 2–4.5 V, b) voltage profiles at a limited capacity of 500 mAh g^−1^, and cycling performance at limited capacities of c) 500 andd) 1000 mAh g^−1^.

**Figure** **3**a gives the voltage profiles of Li–O_2_ batteries with the G/Au‐NP/Au‐NS cathode at a cutoff voltage of 2–4.5 V under 400 mA g^−1^ (0.24 mA cm^−2^). A high discharge capacity of 3347 mAh g^−1^ is obtained with a flat discharge plateau. By contrast, Li–O_2_ battery with bare graphene delivers a discharge capacity of only 89 mAh g^−1^ at a smaller current density of 200 mA g^−1^ (Figure S4, Supporting Information), indicating that graphene itself contributes minor to the catalytic activity of G/Au‐NP/Au‐NS, and thus can only be considered as the conducting support for ORR/OER. Therefore, the current density and specific capacity of the batteries were calculated based on the weight of Au. Figure 3b,c shows the voltage profiles and cycling performance of the G/Au‐NP/Au‐NS‐catalyzed Li–O_2_ battery at a limited capacity of 500 mAh g^−1^. The capacity of 500 mAh g^−1^ can be maintained over 300 cycles at 400 mA g^−1^. In addition, in most of these cycles, the terminal discharge voltage is over 2.5 V, indicative of a low electrode polarization with Li_2_O_2_ deposition. The OER potentials are somewhat higher than expected which may be due to the relatively high current density used and sluggish transport rate of Li ions and oxygen in the presence of the Au film. The batteries were also tested at lower current densities of 100 and 200 mA g^−1^ (Figure S5, Supporting Information). As expected, the OER potentials can be obviously reduced at lower current densities. A stable cycling can last 170 times when the capacity was limited at 1000 mAh g^−1^. A high discharge voltage of over 2.5 V is also observed for the first 120 cycles at 1000 mAh g^−1^, suggesting that an increased Li_2_O_2_ deposition does not cause increased electrode polarization due to the unique electrode design. By contrast, the stable cycling of Li–O_2_ battery with the G/Au‐NP cathode could last only 28 times (Figure S6, Supporting Information).

**Figure 4:**
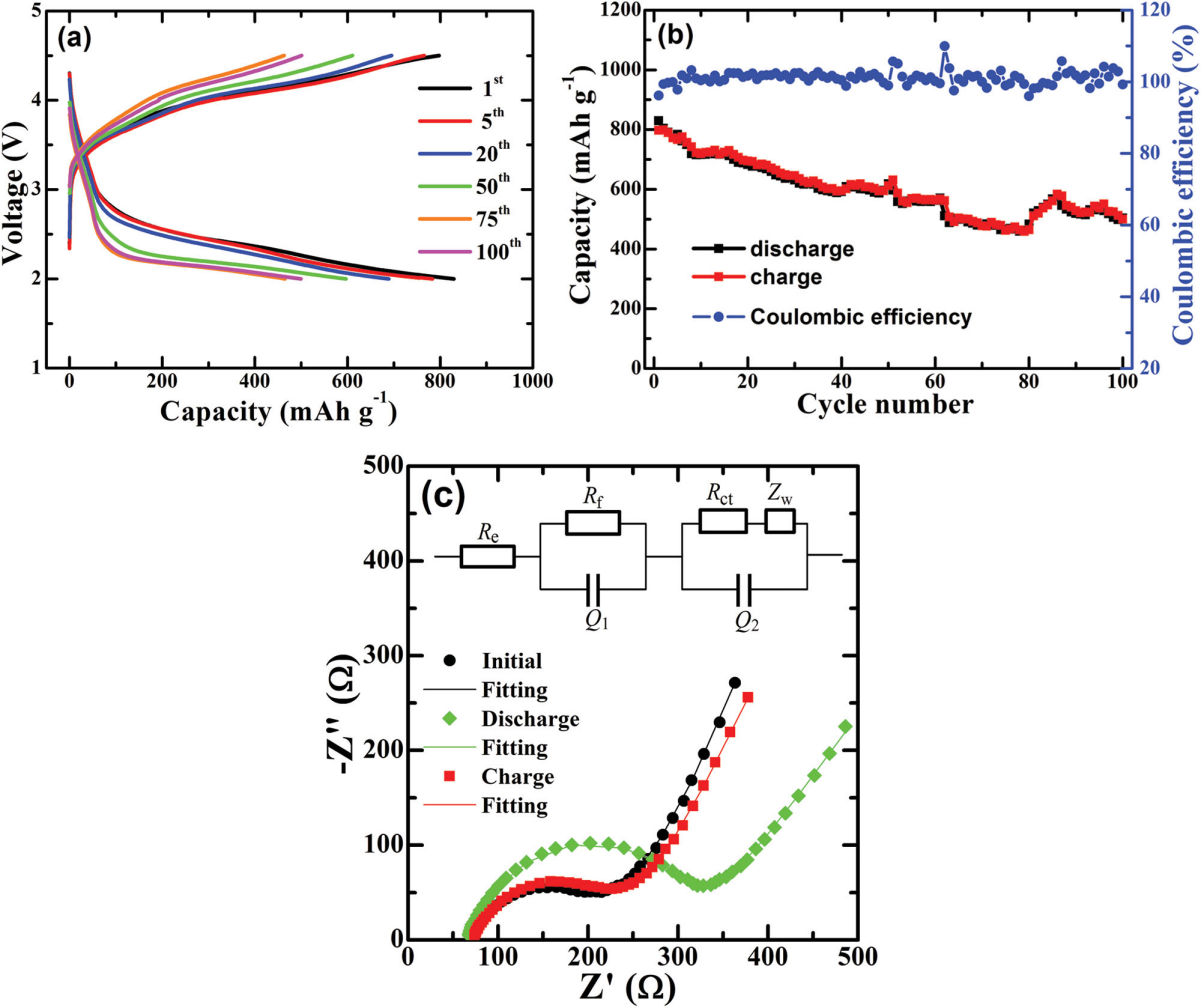
a) Voltage profiles and b) cycle performance of the G/Au‐NP/Au‐NS‐catalyzed Li–O_2_ battery at a cutoff voltage of 2–4.5 V under 800 mA g^−1^, and c) Nyquist plots and the fittings of the Li–O_2_ battery at different charge–discharge states (1000 mAh g^−1^).

**Figure** **4**a,b demonstrates the voltage profiles and cycling performance of Li–O_2_ battery with the G/Au‐NP/Au‐NS cathode in a cutoff voltage of 2–4.5 V under 800 mA g^−1^ (0.48 mA cm^−2^). Note that in this rigorous cycling mode, the Li–O_2_ battery can still exhibit a stable cycling. After 100 cycles, a capacity over 500 mAh g^−1^ is retained. The coulombic efficiency is close to 100% during cycling which suggests the reversible growth/decomposition of Li_2_O_2_. This means that the side reactions related to electrolyte^61^ or carbon^40^, ^41^, ^42^ are not significant. Previous reports showed that carbon^30^, ^41^ or noble metals^34^, ^62^ could catalytically decompose the electrolytes. As shown in Figure S7 (Supporting Information), when tested in pure argon, the battery shows a low capacity with a rapid voltage increase upon charge and a rapid voltage decrease upon discharge. It suggests that the G/Au‐NP/Au‐NS electrode displays minor catalytic effect for electrolyte decomposition. Electrochemical impedance spectroscopy (EIS) was used to understand the excellent catalytic activity of G/Au‐NP/Au‐NS. The Nyquist plots at different states are shown in Figure 4c. The plots are fitted using an equivalent circuit (inset in Figure 4c) and the fitting results are summarized in Table S1 (Supporting Information). In the circuit, *R*_e_ denotes ohm resistance of the battery components, *R*_f_ and *Q*_1_ correspond to solid‐state‐electrolyte resistance and relax capacitance, *R*_ct_ and *Q*_2_ represent charge transfer resistance and double layer capacitance, and *Z*_w_ is related to the bulk diffusion of Li ions.^63^ Note that the increase in *R*_ct_ is not significant after discharge, implying that Li_2_O_2_ deposition does not cause obvious electrode deactivation, agreeing well with the electrochemical tests. The reduction of *R*_ct_ after recharge indicates the sufficient decomposition of Li_2_O_2_.

**Figure 5:**
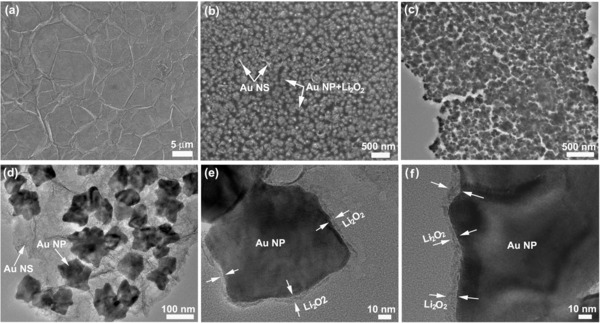
a) SEM image of the discharged G/Au‐NP/Au‐NS electrode on Ni (1000 mAh g^−1^), b) enlarged view of (a), c) TEM image of Au‐NP/Au‐NS exfoliated from the discharged G/Au‐NP/Au‐NS electrode, d) enlarged view of (c), and e,f) HRTEM images showing the Li_2_O_2_ on the surface of Au NPs.

To further clarify the superior catalytic performance of the G/Au‐NP/Au‐NS electrode, SEM and TEM observations were conducted on the discharged electrodes. As shown **Figure** **5**a, the surface profiles are maintained after discharge without the formation of any cracks. The enlarged image in Figure 5b shows that the discharged Au NPs are still well separated with no large Li_2_O_2_ particles formed. Besides, the Au NSs are visible after discharge, indicating the structural integrity of the G/Au‐NP/Au‐NS electrode. The transparent nature of the Au NSs implies that both interior and exterior surfaces of the Au NSs are Li_2_O_2_ free. In contrast, for the G/Au‐NP electrode, large Li_2_O_2_ particles or particles aggregations form after discharge (Figure S3b, Supporting Information). TEM images in Figure 5c,d confirm that the morphologies of both Au NPs and Au NSs were well preserved after discharge, and that no Li_2_O_2_ particles grew on the surface of Au NSs. HRTEM images in Figure 5e,f clearly reveal that the surface of the Au NPs is covered with a uniform and thin Li_2_O_2_ layer with a thickness below 10 nm. Li 1s and O 1s XPS (Figure S8a,b, Supporting Information) indicate that the dominant discharge product is Li_2_O_2_ although a small amount of Li_2_CO_3_ also forms. The formation of Li_2_CO_3_ can be attributed to the decomposition of electrolyte. Note that repeated cycling does not lead to the accumulation of Li_2_CO_3_ obviously as seen in Figure S8 (Supporting Information), which can explain the good cycling performance of the battery. As seen in Figure S9 (Supporting Information), the battery with G/Au‐NP/Au‐NS electrode can sustain 245 cycles at 100 mA g^−1^, corresponding to a long working period of up to 102 d, indicating that increasing working time does not lead to obvious Li_2_CO_3_ accumulation and that the electrolyte is relatively stable during cycling. It should be noted that although Li_2_O_2_ and Li_2_CO_3_ can be qualitatively detected by XPS, the quantitative information regarding the efficiency of Li_2_O_2_ formation needs more efficient analysis tool such as differential electrochemical mass spectrometry system.^35^ Importantly, Li_2_O_2_ can grow along the surface profiles of the Au NPs, which clearly indicates that Au NPs do catalyze the confined and conformal growth of Li_2_O_2_. This growth behavior of Li_2_O_2_ is favorable considering the facts that the contact between Li_2_O_2_ (or LiO_2_) with graphene can be minimized (Figure S8c, Supporting Information), and that the volume expansion of electrode with Li_2_O_2_ accumulation can be largely avoided. In addition, Li_2_O_2_ with a thin‐layer structure can be easily decomposed upon recharge.^18^, ^63^, ^64^

**Figure 6:**
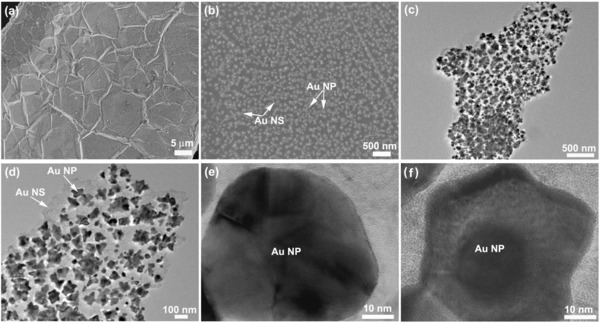
a) SEM image of the recharged G/Au‐NP/Au‐NS electrode on Ni, b) enlarged view of (a), c) TEM image of Au‐NP/Au‐NS exfoliated from the recharged G/Au‐NP/Au‐NS electrode, d) enlarged view of (c), and e,f) HRTEM images of the Au NPs exfoliated from the recharged G/Au‐NP/Au‐NS electrode.

In the G/Au‐NP/Au‐NS electrode, graphene participates in the catalytic reactions by providing the conducting channels,^65^, ^66^ although its catalytic activity is weak as mentioned above. The poor catalytic activity of Au NSs for Li_2_O_2_ growth may be due to the insufficient electron transfer since the Au NPs that bridge graphene with Au NSs are covered with insulating Li_2_O_2_. In this case, the Au NSs may act as the separator to reduce the contact of Li_2_O_2_ (or LiO_2_) with electrolyte, and as the fixer to stabilize the G/Au‐NP electrode. This can explain the considerably improved cycling stability of the G/Au‐NP/Au‐NS‐catalyzed Li–O_2_ battery compared with the G/Au‐NP‐catalyzed battery. SEM images in **Figure** **6**a,b show that the microstructure of the G/Au‐NP/Au‐NS electrode can be retained after the recharge process. The decomposition of Li_2_O_2_ upon recharge can be confirmed by Li 1s and O 1s XPS (Figure S8a,b, Supporting Information). In contrast, Li_2_CO_3_ is remained after charge, suggesting that the decomposition of Li_2_CO_3_ is difficult at the applied voltage (Figure S8b–d, Supporting Information). It was also noticed that the ether peak at around 286. 3 eV appears after the first discharge, and its intensity increases with cycling, suggesting increased amount of decomposition products although the tetraethylene glycol dimethyl ether (TEGDME) electrolyte is relatively stable. Similar result was found in other work using TEGDME as electrolyte.^67^, ^68^ No obvious cracks were generated in Au NSs after recharge, indicating the robustness of the Au NSs. As shown in Figure 6c,d, no aggregation of Au NPs occurs because of the immobilization effect of the Au NSs, ensuring the durability of catalytic activity of the electrodes and long cycle life of the batteries. HRTEM images in Figure 6e,f show that the surface substance is almost removed after charge, indicative of reversibility of formation/decomposition of Li_2_O_2_. The results indicate that controlled growth of Li_2_O_2_ can be achieved by optimizing catalytic electrode, and that the architecture and component are of equal importance in order to realize high performance of Li–O_2_ batteries.

## 3 Conclusion

In summary, a highly efficient catalytic cathode of G/Au‐NP/Au‐NS was prepared by a facile impregnation approach in ice bath. In this electrode, Li_2_O_2_ realizes the conformal growth and crystallizes into thin‐layer form on the surface of Au NPs, graphene supplies the conducting channels for ORR/OER, and Au NSs stabilizes the G/Au‐NP structure and reduces the contact of Li_2_O_2_ (or LiO_2_) with electrolyte. The unique architecture of the G/Au‐NP/Au‐NS electrode enables controlled Li_2_O_2_ growth, easy Li_2_O_2_ decomposition, and reduced side reactions, leading to excellent electrochemical properties of Li–O_2_ batteries. At a limited capacity of 500 mAh g^−1^, the battery can sustain a stable cycling over 300 times at 400 mA g^−1^. The Li–O_2_ battery can maintain a capacity of 500 mAh g^−1^ after 100 cycles at 800 mA g^−1^ when it was tested in a full charge/discharge mode in the cutoff voltage of 2–4.5 V. The results show that the controlled growth of Li_2_O_2_ can be realized by optimizing the electrode design and that both architecture and component of the electrodes are important to achieve high performance of Li–O_2_ batteries.

## 4 Experimental Section

*Electrodes Preparation and Characterization*: Graphene was deposited on Ni foam by a CVD method as described previously.^63^ The loading of graphene on Ni is 0.6 mg cm^−2^. The Ni‐supported G/Au‐NP/Au‐NS electrodes were prepared by a solution impregnation method. Briefly, the graphene‐loaded Ni foam pieces were immersed in a beaker containing HAuCl_4_·3H_2_O aqueous solution (0.16 mg mL^−1^) and the beaker was placed in an ice bath for 3 h. The Ni‐supported G/Au‐NP/Au‐NS electrodes were then rinsed with distilled water, dried at 60 °C for 10 h in vacuum and heated at 300 °C for 2 h in argon. For comparison, the Ni‐supported G/Au‐NP electrodes were also prepared by a similar route with the impregnation step performed at room temperature. The loading of Αu on graphene is 0.5–0.6 mg cm^−2^. The materials deposited on Ni foam were checked by XRD on a Rigaku D/Max‐2550pc diffractometer with Cu K_α_ radiation (*λ* = 1.541 Å). The structure of graphene on Ni was analyzed by Raman spectrum on a Jobin‐Yvon Labor Raman HR‐800 system with 514.5 nm Ar‐ion laser. The morphologies of the pristine, discharged and recharged electrodes were observed by field‐emission SEM on an S‐4800 microscope. The microstructure of electrode components exfoliated from the electrodes was characterized by TEM on a JEM 2100F microscope. The chemical states of the elements in the electrodes were examined by XPS on a Kratos Axis Ultra‐DLD spectrometer with Al K_α_ radiation (*hν* = 1486.6 eV). The electrodes after cycling were carefully handled before the XPS measurements. First, the batteries after charge or discharge were dissembled in the argon‐filled glove box. The electrodes were then washed by 1,2‐dimethoxyethane (DME) three times to remove TEGDME and LiClO_4_ sufficiently on the electrode surface. The DME was then removed by resting the electrodes in the small chamber of the glove box under evacuation for 15 min. After that, the electrodes were fixed on the XPS holder and sealed in a plastics box under Ar atmosphere in the glove box. For XPS tests, the electrodes were taken out from the box and transferred to the XPS chamber as quickly as possible (within 2 min). The XPS chamber was then quickly evacuated.

*Li–O_2_ Batteries Assembly and Electrochemical Measurements*: Coin‐type Li–O_2_ batteries were assembled in an argon‐filled glove box using Li foils as anodes, Ni‐supported G/Au‐NP/Au‐NS (or graphene, G/Au‐NP) as cathodes (0.36 cm^2^), Celgard C480 porous films as separators, and 1 m LiClO_4_ in TEGDME (Sigma‐Aldrich, treated with molecular sieve before use) as electrolyte. The cathodes were dried at 80 °C under vacuum for 10 h prior to assembly of the batteries. The assembled batteries were then purged with O_2_ for 10 min and stayed at open voltage circuit for 5 h to achieve an equilibrium. Galvanostatic cycling was conducted on a Neware battery cycler (Shenzhen, China) over a voltage range of 2.0–4.5 V (vs Li/Li^+^). The specific capacity (mAh g^−1^) and current density (mA g^−1^) of the G/Au‐NP/Au‐NS and G/Au‐NP electrodes were calculated based on the weight of Au. The discharge and charge are referred to lithiation and delithiation, respectively. EIS was recorded on a VersaSTAT3 electrochemistry workstation (Princeton Applied Research) by applying an ac voltage of 10 mV amplitude in a frequency range from 10^−2^ to 10^5^ Hz. The electrochemical tests were all carried out at room temperature.

## Supporting information

As a service to our authors and readers, this journal provides supporting information supplied by the authors. Such materials are peer reviewed and may be re‐organized for online delivery, but are not copy‐edited or typeset. Technical support issues arising from supporting information (other than missing files) should be addressed to the authors.

Supplementary
