# Supplementary material for: High‐Performance Li–O2 Batteries with Controlled Li2O2 Growth in Graphene/Au‐Nanoparticles/Au‐Nanosheets Sandwich
Source: Adv Sci (Weinh). 2016 Apr 28;3(10):1500339. doi: 10.1002/advs.201500339 (PMC5095780; doi:10.1002/advs.201500339)
Supplement: Supplementary file 1 — Supplementary [file ADVS-3-0l-s001.pdf]

Copyright WILEY-VCH Verlag GmbH & Co. KGaA, 69469 Weinheim, Germany, 2016.

ADVANCED SCIENCE
Open Access

Supporting Information

for *Adv. Sci.,* DOI: 10.1002/advs.201500339

High-Performance Li–O_2 Batteries with Controlled Li_2O_2 Growth in Graphene/Au-Nanoparticles/Au-Nanosheets Sandwich

Guoqing Wang, Fangfang Tu, Jian Xie, Gaohui Du, Shichao Zhang, Gaoshao Cao, and Xinbing Zhao*

Supporting Information

High-Performance Li–O_2 Batteries with Controlled Li_2O_2 Growth in Graphene/Au-Nanoparticles/Au-Nanosheets Sandwich

Guoqing Wang, Fangfang Tu, Jian Xie,[*] Gaohui Du, Shichao Zhang, Gaoshao Cao, and Xinbing Zhao

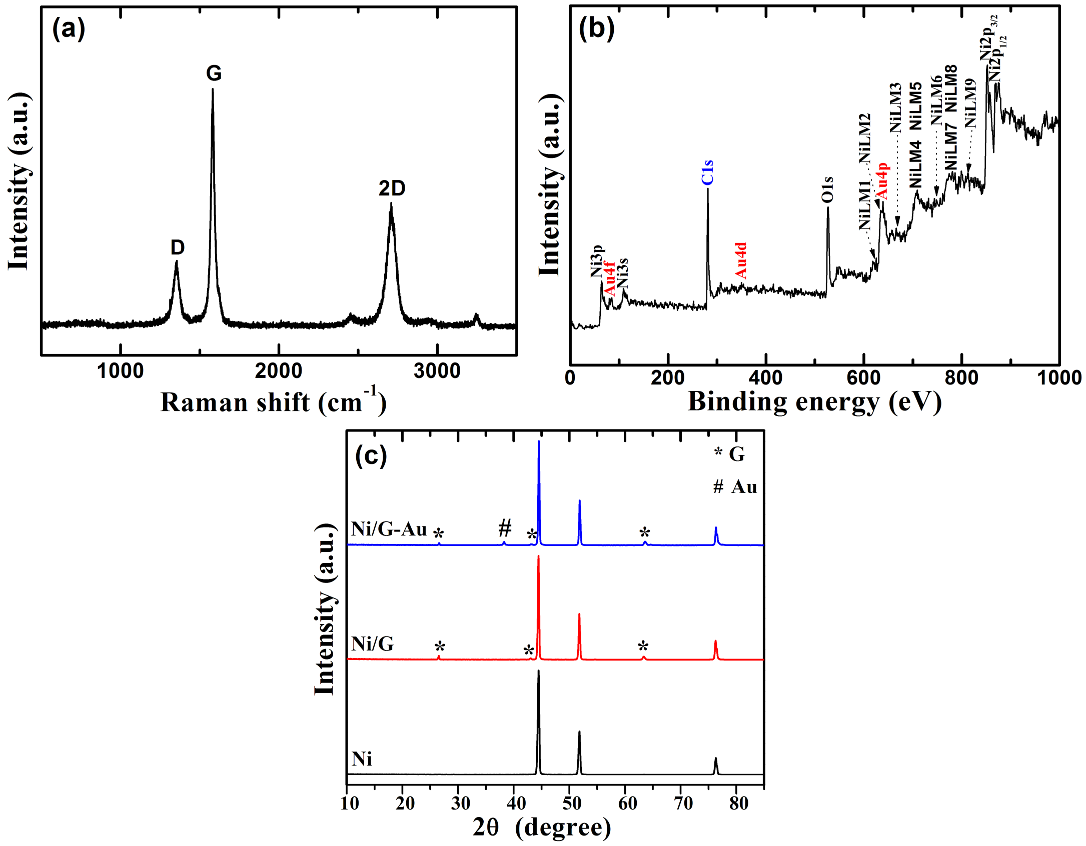

Figure S1. (a) Raman spectrum of the graphene on Ni foam, (b) XPS survey and (c) XRD patterns of the G/Au-NP/Au-NS electrode on Ni foam.

E-mail: xiejian1977@zju.edu.cn; Fax: +86-571-87951451; Tel: +86-571-87951451

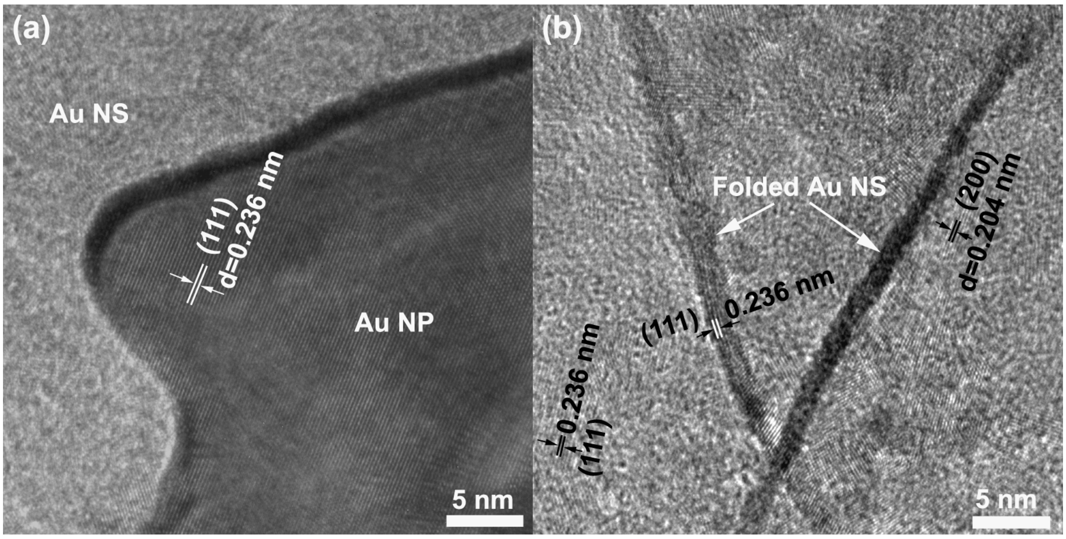

Figure S2. HRTEM images of (a) Au NP and (b) Au NS.

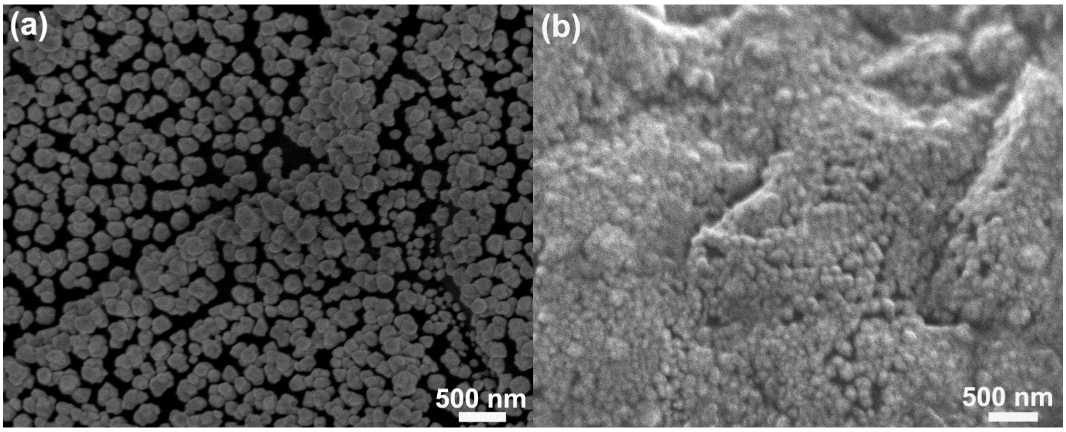

Figure S3. SEM images of the G/Au-NP electrode on Ni foam prepared at room temperature at (a) pristine and (b) discharge states.

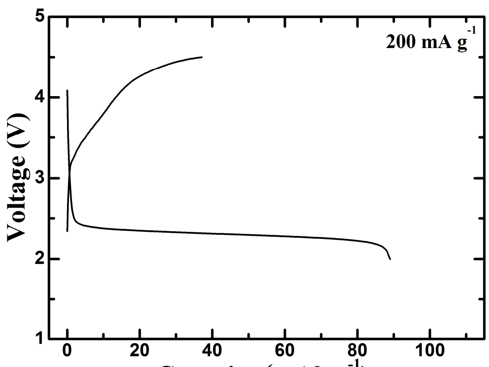

Figure S4. Voltage profiles of the Li−O_2 battery with bare graphene as catalyst.

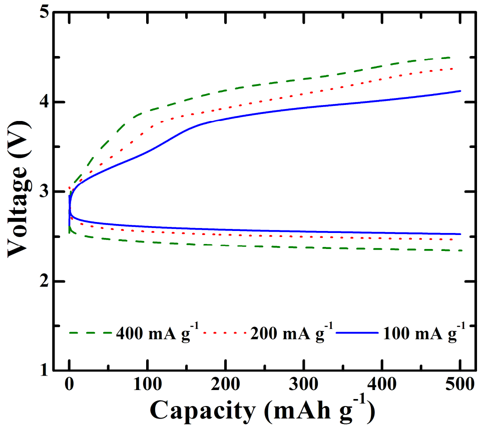

Figure S5. Voltage profiles of the Li–O_2 battery with G/Au-NP/Au-NS cathode at a current density of 100 and 200 mA g^{-1} with a limited capacity of 500 mAh g^{-1}.

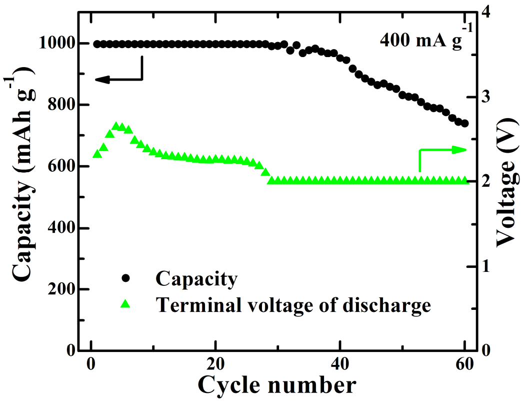

Figure S6. Cycling performance of the G/Au-NP-catalyzed Li–O_2 battery at a limited capacity of 1000 mAh g^{-1}.

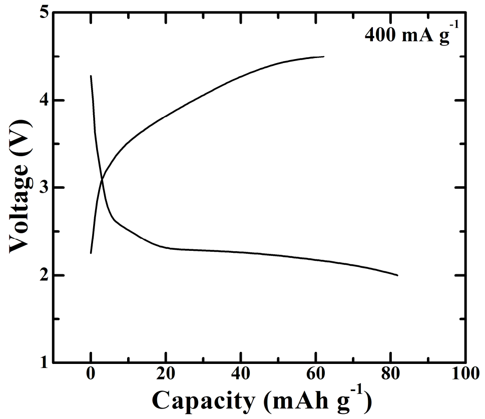

Figure S7. Voltage profiles of the battery tested in pure Ar.

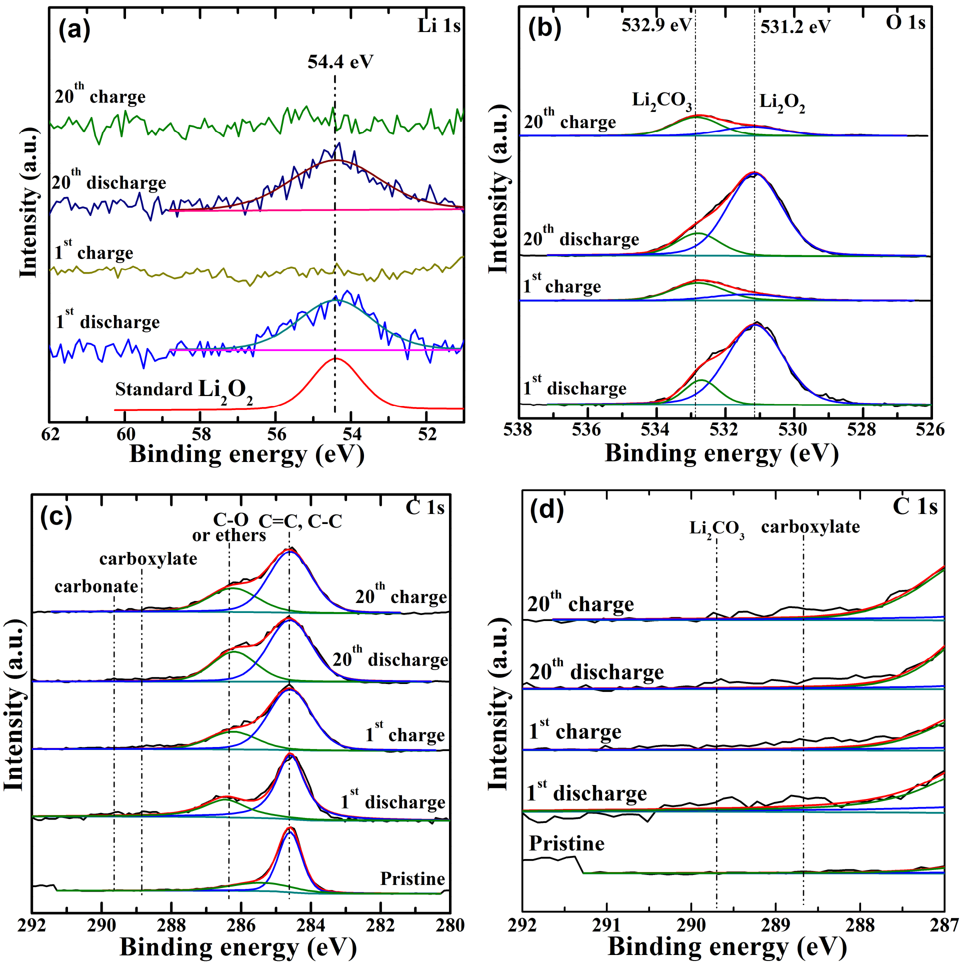

Figure S8. (a) Li 1s, (b) O 1s and (c, d) C 1s XPS of the discharged and re-charged G/Au-NP/Au-NS electrodes. Figure S8d is the enlarged view of Figure S8c at 287–292 eV. The peak at 54.4 eV in Figure S8a and the peak at 531.2 eV in Figure S8b indicate the formation of Li_2O_2, and the peak at 532.9 eV in Figure S8b indicates the formation of Li_2CO_3.[1] In Figure S8c, the C–O or ether peak at 286.3 eV originates from the electrolyte decomposition,[2,3], and its intensity increases with cycling indicating increased amount of decomposition products. Due to strong C1s peak of graphene, the C1s peak of Li_2CO_3 and carboxylate, originating from the reaction between Li_2O_2 (or LiO_2) and carbon (graphene) or electrolyte,[4–7] is not obvious in Figure S8c, but they can still be seen in the enlarged view of Figure S8c (Figure S8d), agreeing with the Li 1s and O 1s XPS tests.

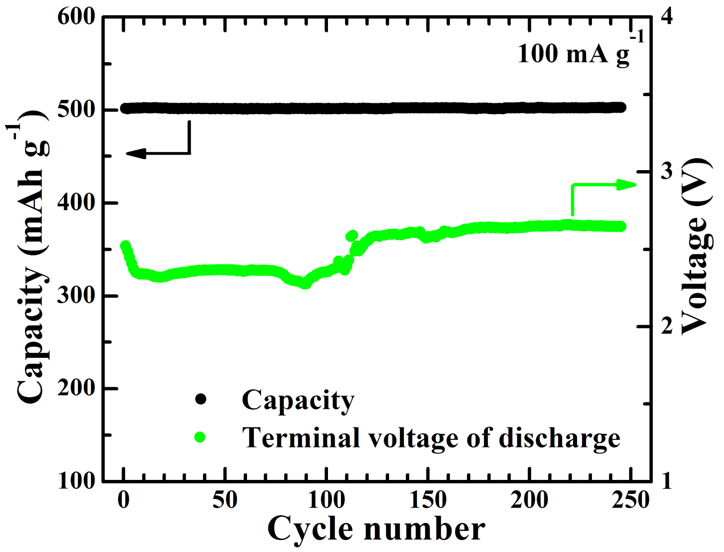

Figure S9. Cycling performance of Li–O_2 battery with G/Au-NP/Au-NS cathode at a current density of 100 mA g^{-1} and a limited capacity of 500 mAh g^{-1}.

Table S1 Fitting results of the Nyquist plots using the equivalent circuit.

Sample	R_e (Ω)	R_f (Ω)	Q_1		R_{ct} (Ω)	Q_2	
			Y	n		Y	n
Initial	67.8	66.2	4.1×10^{-5}	0.55	127.1	7.1×10^{-6}	0.77
After discharge	64.8	103.7	8.5×10^{-6}	0.75	148.0	2.2×10^{-6}	0.98
After charge	67.2	101	2.7×10^{-4}	0.47	111.3	4.9×10^{-6}	0.85

References

[1] Y. Q. Chang, S. M. Dong, Y. H. Ju, D. D. Xiao, X. H. Zhou, L. X. Zhang, X. Chen, C. Q. Shang, L. Gu, Z. Q. Peng, G. L. Cui, *Adv. Sci.* **2015**, *2*, 1500092.

[2] B. G. Kim, S. Kim, H. Lee, J. W. Choi, *Chem. Mater.* **2014**, *26*, 4757−4764.

[3] I. C. Jang, S. Ida, T. Ishihara, *Chemelectrochem*, **2016**, *2*, 1380–1384.

[4] B. D. McCloskey, A. Speidel, R. Scheffler, D. C. Miller, V. Viswanathan, J. S. Hummelshøj, J. K. Nørskov, A. C. Luntz, *J. Phys. Chem. Lett.* **2012**, *3*, 997−1001.

[5] M. M. Ottakam Thotiyl, S. A. Freunberger, Z. Q. Peng, P. G. Bruce, *J. Am. Chem. Soc.* **2013**, *135*, 494−500.

[6] B. M. Gallant, R. R. Mitchell, D. G. Kwabi, J. G. Zhou, L. Zuin, C. V. Thompson, Y. Shao Horn, *J. Phys. Chem. C* **2012**, *116*, 20800−20805.

[7] D. M. Itkis, D. A. Semenenko, E. Y. Kataev, A. I. Belova, V. S. Neudachina, A. P. Sirotina, M. Hävecker, D. Teschner, A. Knop Gericke, P. Dudin, A. Barinov, E. A. Goodilin, Y. Shao Horn, L. V. Yashina, *Nano Lett.* **2013**, *13*, 4697−4701.
